# Protective effect of isoliquiritigenin in amiodarone‐induced damage of human umbilical vein endothelial cells

**DOI:** 10.1002/iid3.1094

**Published:** 2023-11-28

**Authors:** Jin‐Li Guo, Xiang Han, Xian‐Yan Yan, Juan‐Juan Wang, Ya‐Qiong Chang, Bei‐Lei Zhang, Xiu‐Juan Guo

**Affiliations:** ^1^ Department of Nursing Second Hospital of Shanxi Medical University Taiyuan China; ^2^ School of Nursing Shanxi Medical University Taiyuan China

**Keywords:** amiodarone, angiogenesis, endothelial cells, isoliquiritigenin, phlebitis

## Abstract

**Objective:**

Amiodarone (AM) is a drug commonly used in patients with ventricular arrhythmias. It can damage vascular endothelial cells and easily cause phlebitis. At present, the prevention and treatment of phlebitis induced by the use of AM is not clear due to the lack of corresponding primary research. Isoliquiritigenin (ISL) has an anti‐inflammatory effect, but until now, has not been explored much in the field of research in primary care nursing. The purpose of this study is to investigate the efficacy and mechanism of action of ISL in treating phlebitis induced by AM.

**Methods:**

In our study, we used human umbilical vein endothelial cells (HUVECs) that were divided into three groups: the NC group (normal), the AM group (AM 30 μmol/L for 24 h), and the ISL pretreatment group (isoliquiritigenin 10 μmol/L after 1 h of pretreatment with amiodarone for 24 h). We used CCK‐8 to detect cell proliferation, cell scratch assay to detect the migration capability of cells, flow cytometry to measure apoptosis, angiogenesis assay to check the total length and total branches of angiogenesis, and PCR and WB to detect the expression of PCNA, casepase‐3, and VEGFA. WB was used to detect NF‐κBp65 and p‐NF‐κBp65 expression.

**Results:**

Compared with the AM group, the ISL pretreatment promoted cell proliferation and migration, inhibited cell apoptosis, increased the total length and total branches of angiogenesis, and downregulated p‐NF‐κBp65 expression.

**Conclusion:**

ISL shows promise in the prevention and treatment of clinical phlebitis and can be used as a potential therapeutic drug to prevent phlebitis.

## Graphical abstract

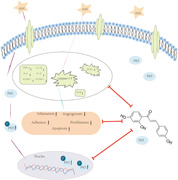

## 1 INTRODUCTION

**Figure 1:**
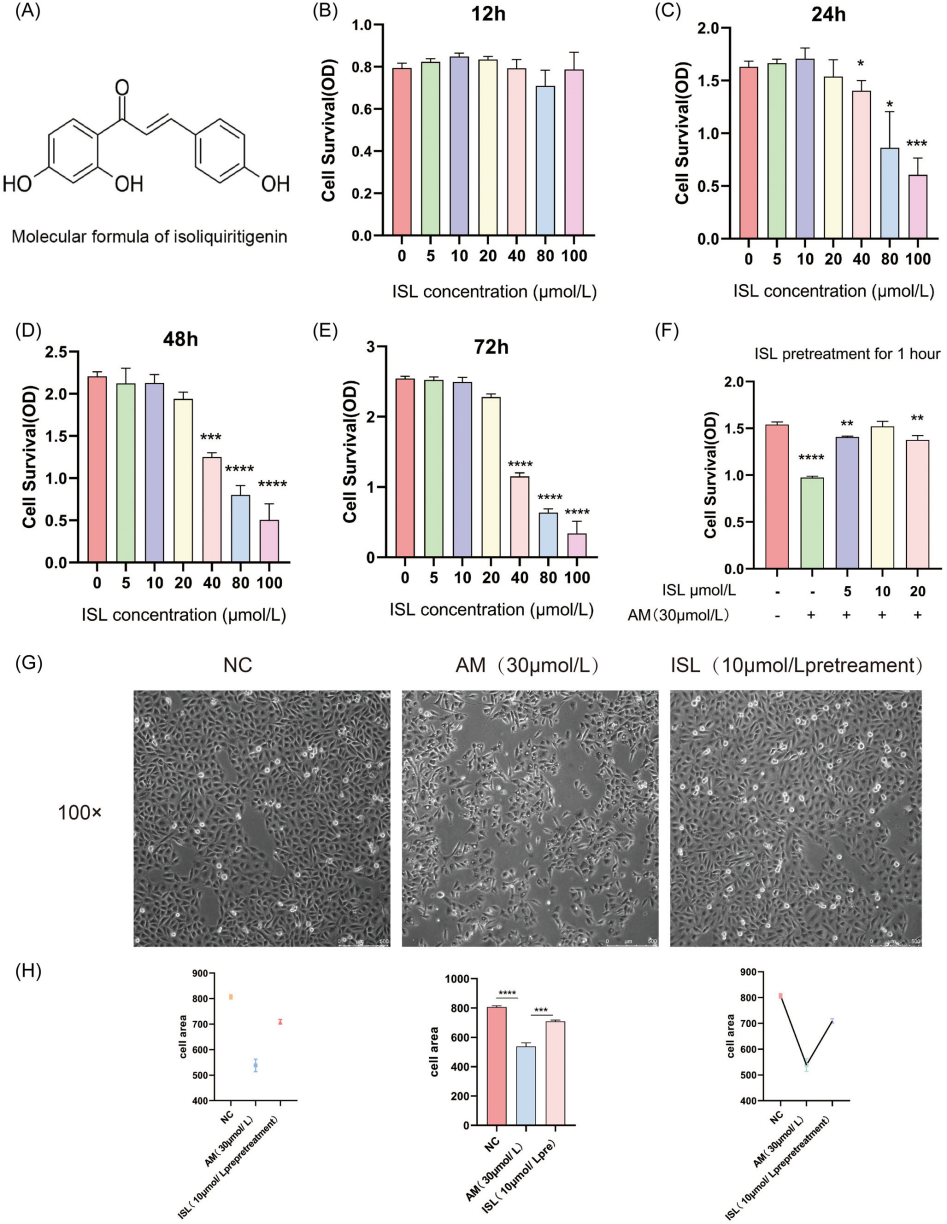
Effects of isoliquiritigenin on the viability and morphology of HUVECs induced by amiodarone. (A) Molecular structure formula of ISL; (B) treatment of HUVEC with different concentrations of ISL for 12 h, cell viability was measured by CCK‐8 method; (C) treatment of HUVEC with different concentrations of ISL for 24 h, cell viability was measured by CCK‐8 method; (D) HUVECs were treated with different concentrations of ISL for 48 h, cell viability was measured by CCK‐8 method; (E) HUVECs were treated with different concentrations of ISL for 72 h, cell viability was measured by CCK‐8 method; (F) pretreatment with different concentrations of ISL(10 μmol/L) for 1 h, and treatment with AM (30 μmol/L) for 24 h. Cell viability was measured by CCK‐8 method; (G) changes of cell state in the NC group, AM group, and ISL pretreatment group. (H) Quantified the area of cells in the normal group, amiodarone group, and isoliquiritigenin pretreatment group under the microscope using ImageJ 8.0 software. The result is expressed as mean ± SD (*n* = 3; ns *p* > .05, *p < .05, **p < .01, ***p < .001, ****p < .0001 vs NC).

Amiodarone (AM) is a drug commonly used in patients with ventricular arrhythmias. However, the low pH value of the AM injection makes it acidic and highly irritating to peripheral blood vessels; and it can damage vascular endothelial cells and easily cause phlebitis.^1^ The incidence of phlebitis caused by peripheral intravenous amiodarone was as high as 8.0–54.5%.^2^ This complication can lead to infection, increased treatment costs, delayed cure, and prolonged hospitalization. Currently, there is a lack of corresponding research in primary care nursing pertaining to the prevention and treatment of phlebitis. It has been found that amiodarone can inhibit the proliferation, inflammatory factors, and increase apoptosis of HUVEC, which leads to the damage of the vascular wall.^3^ ISL (Figure 1A) is a flavonoid compound extracted from the root of licorice. It has broad‐spectrum pharmacological effects such as anti‐inflammatory,^4^ antioxidant stress,^5^ antiviral,^6^ antitumor,^7^ and nerve protection.^8^ There are studies on ISL in macrophage‐induced neuroinflammation,^9^ lipopolysaccharide‐induced inflammation,^10^ membranous glomerulonephritis,^11^ and TNF‐α‐induced HUVEC, but it has not been studied in the field of primary care nursing research. It has been reported that the reduction of angiogenesis can cause vascular damage.^12^ The key steps of angiogenesis are proliferation, migration, and lumen formation.^13^ Studies exploring the proliferation, migration, angiogenesis, and apoptosis of cells found that simvastatin^14^ and salidroside^15^ had a biological protective effect. Therefore, we speculate that the protective effect of ISL is also related to proliferation, migration, apoptosis, and angiogenesis. The NF‐κB signal pathway is a classical anti‐inflammatory pathway. In the model of vinorelbine‐associated phlebitis^16^ and the model of acute glomerulonephritis,^9^ ISL regulates the NF‐κB signaling pathway and plays an anti‐inflammatory role. Hence, we speculate that AM may activate the NF‐κB pathway in HUVECs and ISL can downregulate p‐NF‐κBp65 expression.

Therefore, we propose the following hypothesis: AM activates the NF‐κB signal pathway, which further inhibits cell proliferation and migration, promotes cell apoptosis, inhibits angiogenesis, and then participates in the formation of phlebitis. ISL can inhibit the NF‐κB signal pathway and play a protective role in phlebitis.

Therefore, in this study, based on previous work, we aimed to observe the effect of ISL on amiodarone‐induced vascular proliferation, migration, apoptosis, and angiogenesis by using CCK‐8, scratch test, flow cytometry, angiogenesis, PCR, WB and other experimental techniques in HUVEC cultured in vitro, to explore its effect on this important signal pathway.

## 2 MATERIALS AND METHODS

In this study, we used FBS (11011‐8611), penicillin mixture (P1400‐100), and trypsin (T1320) from SOLEBO in Beijing, Cell Counting Kit‐8 (HY‐K0301) from MCE in the United States, AnnexinFITC/PI apoptosis detection kit (CA1020) from SOLEBO in Beijing, ISL (HY‐N0102) from MCE in the United States, AM was purchased from Shanghai Sanofi company, rabbit anti‐PCNA (A13336), Casepase‐3 (A2156), VEGFA (A12303) rabbit anti‐PCNA was purchased from Wuhan Abclonal company, goat anti‐rabbit secondary antibody (AS014) was purchased from Abclonal company, and matrix adhesive (356234) was purchased from BD company in the United States. The first rabbit anti‐p65 (WL01273b) and pp65 (WL02169) were purchased from Shenyang Wanke Biotechnology Co., Ltd. The nucleoprotein extraction kit (KGP250) was purchased from Jiangsu Kaiji Biological Co., Ltd. Trizol kit (9108) was purchased from Takara Company in Japan, TB Green® Premix Ex Taq™ II (RR820A) was purchased from Takara Company in Japan.

### 2.1 Cell culture and treatment

HUVEC lines were purchased from the Cell Resource Center of Shanghai Institute of Life Sciences. It was cultured in DMEM culture medium containing 10% fetal bovine serum and 1% penicillin streptomycin mixture, placed in a carbon dioxide incubator (37°C, 5% carbon dioxide).

Treatment conditions: NC group: no treatment; AM group: (AM 30 μmol/L treatment for 24 h) prepared according to instructions^3^; ISL pretreatment group: (AM 30 μmol/L for 24 h) at 10 μmol/L ISL pretreatment.

This experiment is a sequential study. The research group conducted a preliminary study on the concentration and time of amiodarone induced damage to human umbilical vein endothelial cells. This study explores the protective effect of isoliquiritigenin on the basis of the previous study.

### 2.2 Determination of cell viability

#### 2.2.1 Determining the optimal concentration of ISL for nontoxic effect on HUVEC

We added HUVEC to 1 hole × 10^4^ inoculated in 96‐well plate and set the ISL concentration gradient. After termination of culture, we added 10 μL CCK‐8 reagent into each well, incubated for 2 h, and checked the absorbance value of each group (at 450 nm wavelength) using the microplate reader. Absorbance value (OD value of each hole minus OD value of zero adjustment hole). We detected the absorbance values at 12 h, 24 h, 48 h, and 72 h.

#### 2.2.2 Identifying the optimal concentration of ISL for the protection of AM‐induced HUVEC

We took the above concentration that is not harmful to normal cells. ISL pretreatment was done for 1 h, amiodarone (30 μmol/L) for 24 h. The same method mentioned earlier was used to detect OD value.

### 2.3 Scratch test

HUVEC was evenly inoculated into 6‐well plates, and the cells were treated as the NC group, the AM group, and the ISL pretreatment group. Using a sterile gun head (10 μL), we drew three straight lines from top to bottom at the bottom center of each hole of the 6‐well plate, causing scratches. The cells in each group were gently washed with PBS solution, and incubated with serum‐free medium for 6 h, 12 h, and 24 h in a cell incubator. Image J 8.0 was used to analyze the scratch area at 0 h, 6 h, 12 h, and 24 h.

### 2.4 Flow type apoptosis test

HUVEC was evenly inoculated into 6‐well plates. Cells were treated as the NC group, the AM group, and the ISL pretreatment group. Cells were digested with trypsin without EDTA, placed in 1.5 ml Ep tube, washed twice with PBS precooled at 4°C. We collected 1 × 10^5^ cells and placed them in flow tubes, added 100 μL blinding buffer resuspension cells per tube; Then add 5 μL AnnexinV‐FITC, and incubate it in the dark with tin foil for 5 min at room temperature, then add 5 μL PI solution into each tube, and then tested it on the machine.

### 2.5 In vitro luminal formation experiment

HUVEC was evenly inoculated into 6‐well plates, and cells were treated as the NC group, the AM group, and the ISL pretreatment group. We used a precooled gun tip to evenly lay 50 μL per hole of matrix adhesive in the precooled 96‐hole plate. We placed it in a 37°C constant temperature incubator, and let it stand for 30 min until it solidified. Then, the prepared cells were digested, we added 100 μL cell suspension into each well, with cell density controlled at 1 × 10^4^. We observed the formation of blood vessels through microscope at 2 h, 4 h, 6 h, and 24 h, respectively. We analyzed the length of cell tubes and the number of branches of blood vessels using ImageJ 8.0 software.

### 2.6 PCR

HUVEC was evenly inoculated into 6‐well plates, and cells were treated as the NC group, the AM group, and the ISL pretreatment group. We used Trizol reagent to extract total RNA of cells, and detected the D260/D280 value with an ultraviolet spectrophotometer. RNA was reverse‐transcribed into cDNA as per the reverse transcription kit, and GAPDH was used as the internal reference for PCR amplification. The relative mRNA amount was calculated using 2^−△△T^. Details for primer pair and amplification reaction are shown in Table 1.

**Table 1:** Synthesis list of real‐time PCR primer.

Gene	Primer sequence 5′‐3′
IL‐1β	F: GCCAGTGAAATGATGGCTTATT
	R: AGGAGCACTTCATCTGTTTAGG
TNF‐α	F: TGGCGTGGAGCTGAGAGATAACC
	R: CGATGCGGCTGATGGTGTGG
IL‐10	F: GTTGTTAAAGGAGTCCTTGCTG
	R: TTCACAGGGAAGAAATCGATGA
IL‐6	F: CACTGGTCTTTTGGAGTTTGAG
	R: GGACTTTTGTACTCATCTGCAC
GAPDH	F: TGAACGGGAAGCTCACTGG
	R: TCCACCACCCTGTTGCTGTA
ICAM‐1	F: CACAAGCCACGCCTCCCTGAAACCTA
	R: TGTGGGCCTTTGTGTTTTGATGCTA
VCAM‐1	F: TATCTGCATCGGGCCTCACT
	R: AGGAAAAGAGCCTGTGGTGC
VEGFA	F: GGAGTACCCTGATGAGATCGA
	R: CATTTGTTGTGCTGTAGGAAGC
PCNA	F: CCTGCTGGGATATTAGCTCCA
	R: CAGCGGTAGGTGTCGAAGC
Caspase‐3	F: GTGGAGGCCGACTTCTTGTATGC
	R: TGGCACAAAGCGACTGGATGAAC

*Note*: F: forward primer; R: reverse primer.

### 2.7 Western blot

HUVEC was evenly inoculated into 6‐well plates, and cells were treated as the NC group, the AM group, and the ISL pretreatment group. We used the whole protein extraction kit to extract the total protein and then the protein was denaturated. We prepared glue with 10% glue preparation kit, added the protein samples, conducted SDS‐PAGE gel electrophoresis, and transferred it to PVDF membrane, blocked it with 5% skim milk for 1 h, washed it with TBST, and incubated the primary antibody at 4°C overnight. The following main primary antibodies (PCNA 1:1000, casepase‐3 1:1000, VEGFA 1:1000, p65 1:500, pp65 1:500), and then washed thrice with TBST. The goat anti‐rabbit IgG (H + L) was incubated for 1 h, and then washed with TBST. We used the ECL ultra sensitive chemiluminescence kit to prepare the luminous solution according to 1:1, β‐actin was used as the internal parameter, and the protein density value was analyzed using Imagelab.

### 2.8 Statistical analysis

The data were analyzed using Graphpad 8.0. The data of three or more groups were analyzed using one‐way ANOVA, and the data between the two groups were analyzed using *t*‐test. All data were expressed by mean ± standard deviation, *P* ＜ 0.05 was considered as statistically significant.

## 3 RESULTS

### 3.1 Pretreatment with ISL increased the viability of phlebitis cells induced by AM and restored damaged cell morphology

To explore the concentration of ISL with nontoxic effect on HUVEC, the CCK‐8 method was used to select the concentration range of ISL (0–100 μmol/L), respectively for 12 h, 24 h, 48 h, and 72 h (Figure 1B–D). We found that at 12 h, all concentration ranges had no obvious damage to cells (0–20) μmol/L at 24 h, 48 h, and 72 h, there was no obvious damage to cells. Therefore, this concentration range was selected as the ISL concentration with nontoxic effect on cells for subsequent experiments.

In this study, to determine whether ISL can play a role in the proliferation of HUVEC induced by AM, and to find its optimal concentration, we used CCK‐8 to detect the effect of different groups on cell viability before treating HUVEC with AM, ISL (0–20 μmol/L) pretreatment for 1 h. The results showed that the protective concentration of ISL was related to its concentration, the cell viability increased to 93.4%, while AM (30 μmol/L) decreased to 63.2%. (Figure 1F). Through the above experiments, it was finally determined that the pretreatment group of ISL was treated with 30 μmol/L AM for 24 h, and 10 μmol/L ISL was used for 1 h.

We observed the changes of cell morphology in the NC group, the AM group, and the ISL pretreatment group microscopically under low‐power and high‐power microscopes. We found that cells in the AM group had differentiation and increased peripheral burrs, while after pretreatment with ISL, most cells recovered to a short spindle shape. (Figure 1G). Therefore, pretreatment with ISL could restore the morphology of HUVEC induced by AM.

### 3.2 ISL pretreatment increased the migration ability of HUVEC induced by AM and promoted cell proliferation

**Figure 2:**
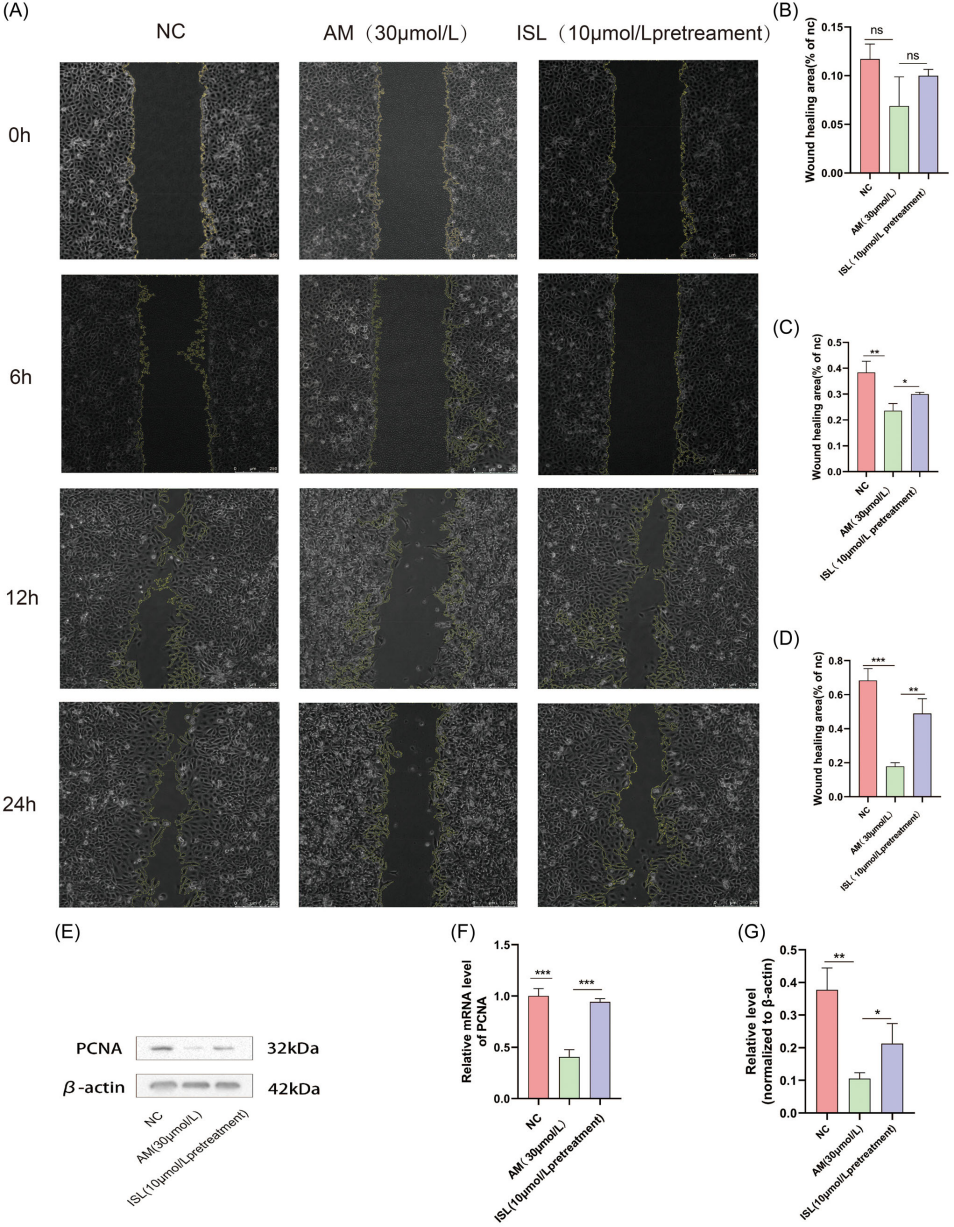
Effect of ISL on migration of HUVECs‐induced by AM. (a) Scratch method was used to detect the influence of the NC group, AM group, and ISL pretreatment group on cell migration at 0 h, 6 h, 12 h, and 24 h, respectively; (b) quantitative wound healing results at 6 h; (c) quantitative wound healing results at 12 h; (d) quantitative wound healing results at 24 h; (e) the expression of PCNA protein in normal group, AM group, and ISL pretreatment group was detected by Western blot; (f) detection of PCNA gene expression in NC group, AM group, and ISL pretreatment group by RT‐PCR; (g) quantification of expression of PCNA protein in normal group, AM group, and ISL pretreatment group. The result is expressed as mean ± SD (*n* = 3; ns *p* > .05, *p < .05, **p < .01, ***p < .001, ****p < .0001 vs AM).

We used the scratch test to evaluate the cell healing at 6 h, 12 h, and 24 h in the NC group, the AM group, and the ISL pretreatment group (Figure 2A) We found that at 6 h, there was no significant difference in the healing ability of the three groups of cells (Figure 2B). At 12 h, the AM group inhibited the migration of HUVEC, while ISL pretreatment promoted the migration of HUVEC (Figure 2C). At 24 h, it could be seen under the microscope that AM group inhibited the migration of HUVEC, while ISL pretreatment promoted the migration of HUVEC (Figure 2D). Therefore, we found that pretreatment with ISL could increase the migration ability of HUVEC induced by AM.

RT‐PCR and WB experiments were used to detect the expression of proliferation related factors (PCNA). We found that that the expression of PCNA gene and protein were decreased in the AM group, while ISL pretreatment significantly increased the expression of proliferation‐related genes and proteins (Figure 2E–G). Therefore, we found that ISL could promote the proliferation of HUVEC‐induced by AM, thereby promoting cell healing.

### 3.3 ISL pretreatment reduced the apoptosis of HUVEC‐induced by AM and downregulated the expression of casease‐3

We used flow cytometry to detect apoptosis in the NC group, the AM group, and the ISL pretreatment group.

In the apoptosis map, cells can be divided into four quadrants. The upper left quadrant represents dead cells, while the lower left quadrant represents normal living cells. The upper right quadrant represents late apoptotic cells, while the lower right quadrant represents early apoptotic cells.

**Figure 3:**
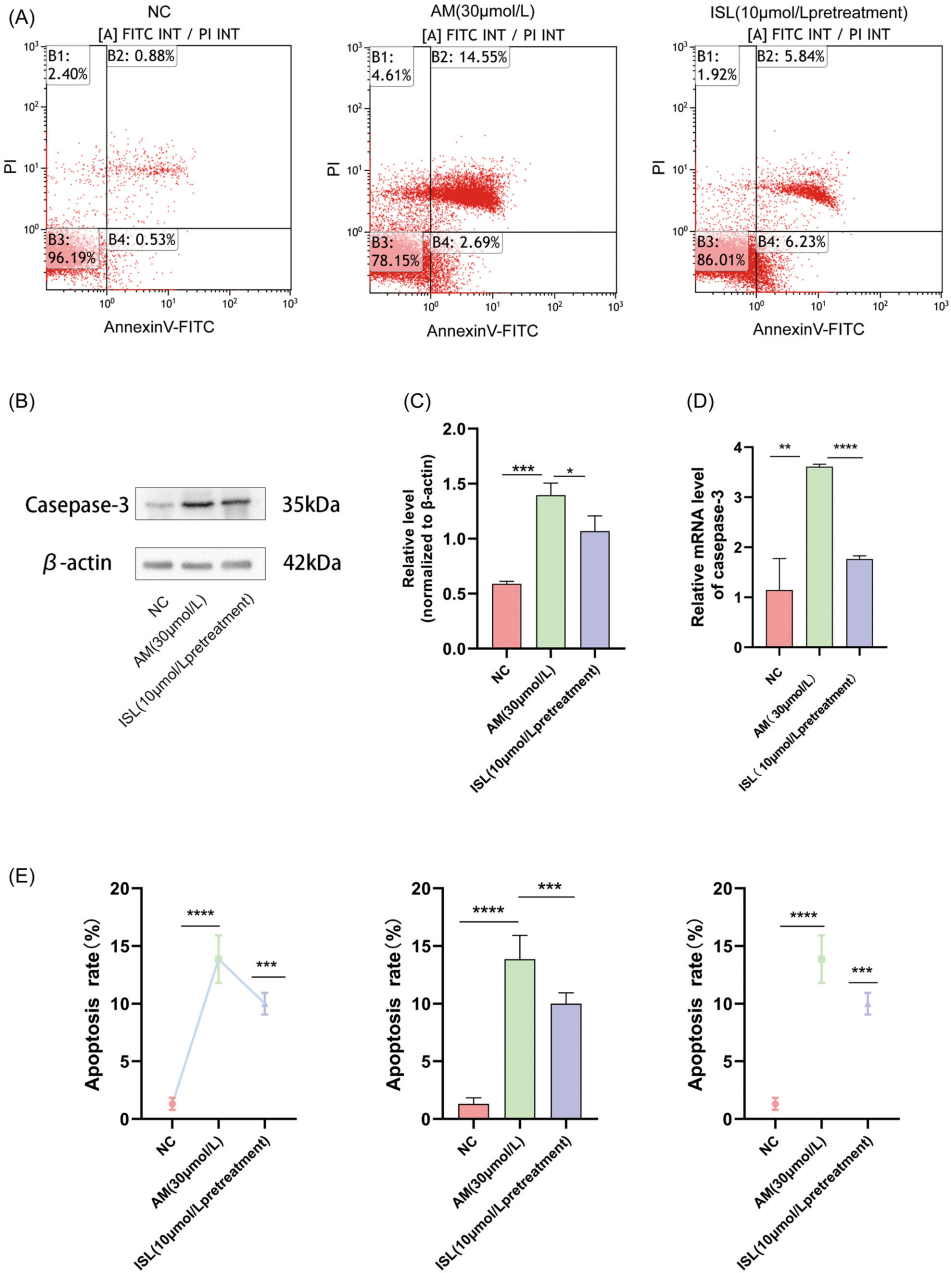
Effect of ISL on AM induced apoptosis of HUVECs. (A) Flow cytometry was used to detect the expression of apoptosis in the NC group, AM group, and ISL pretreatment group; (b) Western blot was used to detect the expression of casease‐3 protein in NC group, AM group, and ISL pretreatment group; (c) quantification of the expression of casapse‐3 protein in NC group, AM group, and ISL pretreatment group; (d) detection of casepase‐3 gene expression in NC group, AM group, and ISL pretreatment group by RT‐PCR; (e) quantification of flow cytometry apoptosis data in NC group, AM group, and ISL pretreatment group. The result is expressed as mean ± SD (*n* = 3; ns *p* > .05, *p < .05, **p < .01, ***p < .001, ****p < .0001 vs AM).

Annexin V FITC/PI experiment showed that the apoptosis rate of the NC group was 3.8%, and that of the AM group was 17.2%. After pretreatment with ISL, the apoptosis rate decreased to 11.1%. (Figure 3A), demonstrating that ISL could reduce the apoptosis of HUVEC induced by AM.

Additionally, we used RT‐PCR and WB assay in this study to detect the expression of caspase‐3. We found that after treatment with AM, the expression of casease‐3 gene and protein were increased, while after pretreatment with ISL, the expression of apoptosis‐related genes and proteins were significantly reduced (Figure 3B‐D). Thus, at the gene and protein levels, ISL could reduce the apoptosis of HUVEC induced by AM.

### 3.4 ISL pretreatment increased vasculogenesis and upregulated vascular endothelial growth factor

**Figure 4:**
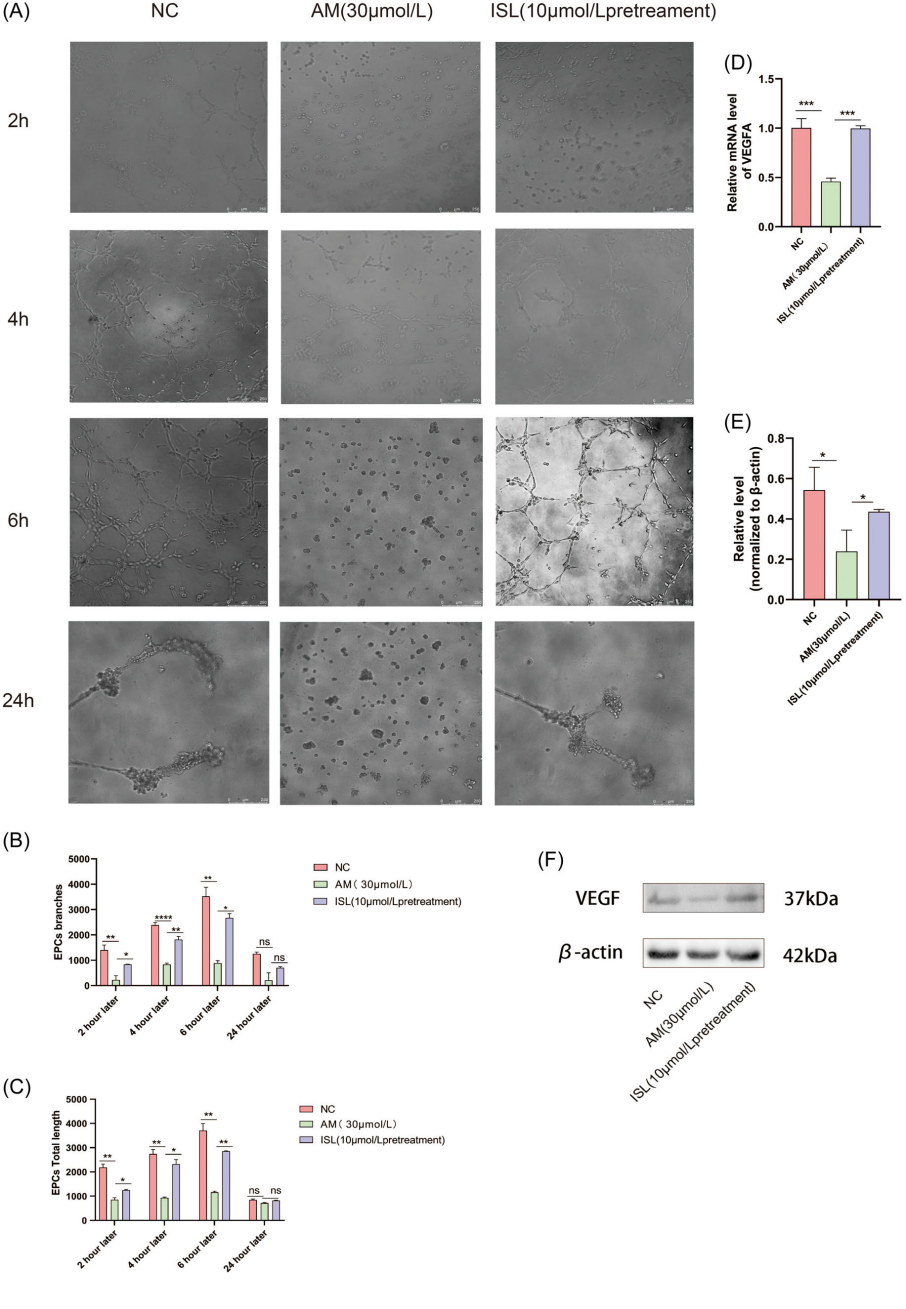
Effect of ISL on AM induced angiogenesis on HUVECs. (a) Matrix glue was used to detect angiogenesis in the NC group, AM group, and ISL pretreatment group at 2 h, 4 h, 6 h, and 24 h, respectively; (b) quantification of the total length of blood vessels in NC group, AM group, and ISL pretreatment group at 2 h, 4 h, 6 h, and 24 h, respectively; (c) quantification of total vascular branches NC group, AM group, ISL pretreatment group at 2 h, 4 h, 6 h, 24 h, respectively; (d) detection of VEGFA gene expression in NC group, AM group, and ISL pretreatment group by RT‐PCR; (e) quantification of expression of VEGFA protein in NC group, AM group, and ISL pretreatment group; (f) results of Western blot for expression of VEGFA protein in NC group, AM group, and ISL pretreatment group. The result is expressed as mean ± SD (*n* = 3; ns *p* > .05, *p < .05, **p < .01, ***p < .001, ****p < .0001 vs AM).

We used the vasculogenesis experiment to investigate the total length and total branches of the vasculogenesis at 2 h, 4 h, 6 h, and 24 h in HUVEC under the microscope (Figure 4A). At 2 h, 4 h, and 6 h, the total length and total number of branches of tubes in the AM group were less, while the total length and total number of branches of vasculogenesis increased after ISL pretreatment. With the increase of time, the total length and total number of branches of blood vessels in different groups increased, while at 24 h, HUVEC was dead, and there was no statistical significance in the length and number of branches of blood vessels among the three groups (Figure 4B–C). Therefore, pretreatment with ISL increased the vasculogenesis of HUVEC induced by AM.

We used RT‐PCR and WB assay in this study to detect the expression of vascular endothelial related factor (VEGFA). We found that AM treatment reduced the expression of VEGFA gene and protein, while ISL pretreatment significantly increased the expression of VEGFA gene and protein (Figure 4D–F). Therefore, at the gene and protein levels, ISL could increase the angiogenesis of HUVEC induced by AM.

### 3.5 The protective effect of ISL on AM‐induced phlebitis may be related to activation of the NF‐κB channel

**Figure 5:**
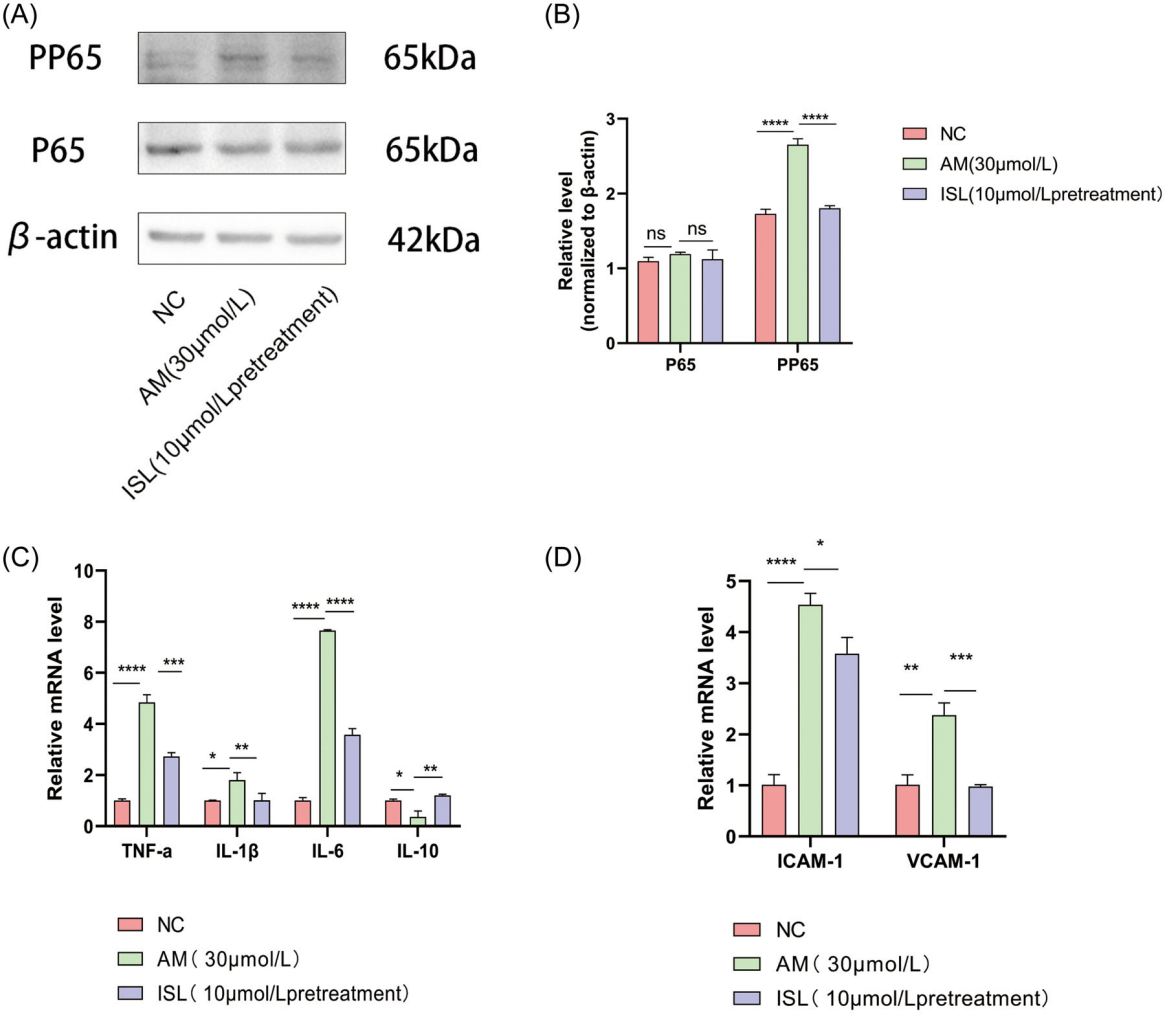
Mechanism of ISL on AM‐induced damage in HUVECs. (A) Results of Western blot for expression of p65, pp65 protein in the NC group, AM group, and ISL pretreatment group; (B) quantification of expression of NF‐κBp65, p‐NF‐κBp65 protein in NC group, AM group, and ISL pretreatment group; (C) detection of TNF‐α, IL‐1β, IL‐6, IL‐10 gene expression in the NC group, AM group, and ISL pretreatment group by RT‐PCR. (D) RT‐PCR results of VCAM‐1, ICAM‐1 gene expression in the NC group, AM group, and ISL pretreatment group. The result is expressed as mean ± SD (*n* = 3; ns *p* > .05, *p < .05, **p < .01, ***p < .001, ****p < .0001 vs AM).

In this study, we used WB experiment to explore factors related to the NF‐κB pathway (NF‐κBp65, p‐NF‐κBp65). We found that the total protein content between different groups of NF‐κBp65 remained unchanged. After AM treatment, p‐NF‐κBp65 protein expression was increased, and after pretreatment with ISL, the expression of the pathway‐related protein p‐NF‐κBp65 was significantly decreased. This result showed that ISL could inhibit the NF‐κB activation of HUVEC induced by amiodarone. After pretreatment with ISL, the expression of the pathway‐related protein p‐NF‐κBp65 was significantly decreased. We further evaluated the related inflammatory factors (TNF‐α, IL‐1 β, IL‐6, IL‐10) and expression of adhesion factors (ICAM‐1, VCAM‐1) (Figure 5C‐D). We found that after treatment with AM, the expression of inflammatory factors (TNF‐α, IL‐1 β, IL‐6) and adhesion factors (ICAM‐1, VCAM‐1) decreased the expression of anti‐inflammatory factor (IL‐10). After ISL pretreatment, the expression of genes related to inflammatory factors and adhesion factors decreased significantly, while the expression of anti‐inflammatory factors increased. The results showed that ISL could play an anti‐inflammatory role on HUVEC induced by AM.

## 4 DISCUSSION

**Figure 6:**
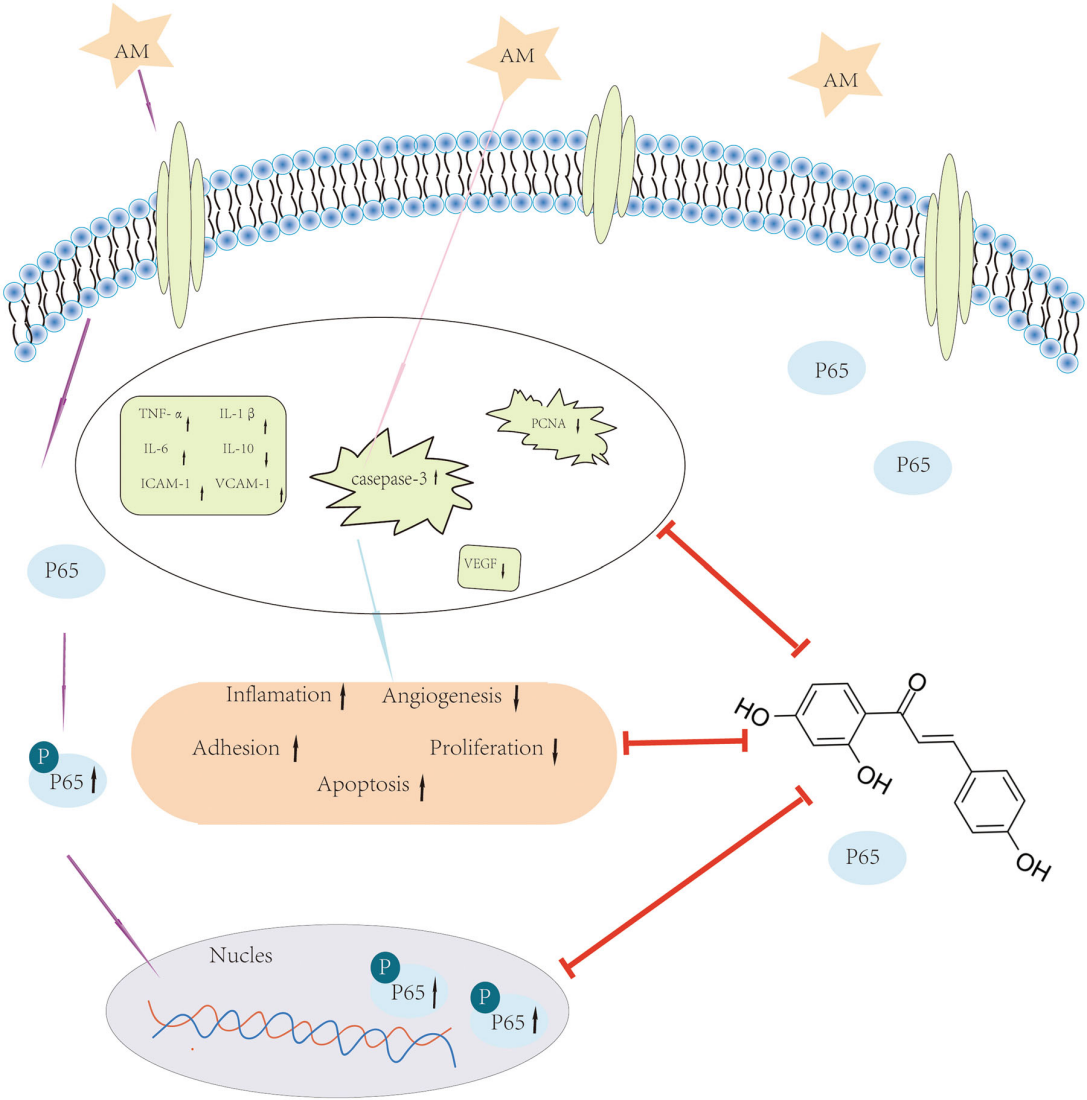
Schematic diagram of the mechanism. Protective effect of ISL in alleviating amiodarone‐induced damage in human umbilical vein endothelial cells (proliferation, migration, apoptosis, and angiogenesis in HUVEC).

In clinical nursing, it is observed that peripheral intravenous infusion of AM can cause blood vessel injury and induce phlebitis, but there are few available studies on its mechanism of action. In this study, we investigated whether ISL has a potential protective effect on AM‐induced phlebitis and its possible mechanism of action. Our research results revealed for the first time that ISL could promote proliferation, promote migration, and inhibit apoptosis of HUVEC induced by AM, and could increase vasculogenesis, which may be partly due to the anti‐inflammatory effect of ISL in downregulating the NF‐κB pathway (Figure 6).

As shown in Figure 1, results of the CCK‐8 experiment in this study found that ISL could increase the cell viability induced by AM. The reduction of cell differentiation under the microscope further confirmed the protective effect of ISL on AM‐induced phlebitis. Proliferation is essential for the formation of new blood vessels.^17^ Our research findings are consistent with the results of an earlier study that found that ISL had an anti‐inflammatory effect on chronic pancreatitis by significantly inhibiting the proliferation and activation of human pancreatic stellate cells.^18^ This confirmed that ISL can play an anti‐inflammatory role in diseases by acting on cell proliferation. At the cellular morphological level, it was observed that HUVEC differentiated after 24 h of treatment with AM. However, cell differentiation was due to the activation of gene regulators and the amplification of intrinsic cell functions.^19^ This may be the reason why AM can induce cell damage and inflammation easily.

Secondly, as shown in Figure 2, the scratch test in this study found that AM treatment of HUVEC for 24 h could significantly inhibit cell migration, while ISL pretreatment significantly improved the migration of HUVEC induced by AM. This experimental result was consistent with the report that mediators promote the migration of HUVEC, thus playing a role in phlebitis caused by AM.^16^ This further confirmed that ISL can play an anti‐inflammatory role on phlebitis by influencing the migration of endothelial cells. Compared with a previous research that only studied the 24‐h scratch experiment results, in this study, we observed the scratch healing rates at 6 h and 12 h, respectively. It was found that at 12 h, ISL had promoted cell migration, but it could only be reflected quantitatively from the data, and the healing of scratches visible to the naked eye under the microscope was not obvious. However, the results at this time cannot be used as a reference, because the time for drug‐induced phlebitis was insufficient. However, surprisingly, ISL started to play a protective role after 12 h.

Then, as shown in Figure 3, flow cytometry, PCR, and WB were used to verify the antiapoptotic effect of ISL on phlebitis induced by AM from three aspects: phenotype, gene, and protein, reducing the damage of endothelial cells, thereby reducing the phlebitis induced by AM. HUVEC apoptosis plays an important role in the formation of inflammation. Some studies have shown that in atherosclerosis,^20^ cell apoptosis is an early warning signal of disease occurrence, enabling endothelial cells to regulate the imbalance of inflammation. Similarly, angiotensin^21^ causes endothelial dysfunction and inflammation by inducing endothelial cell apoptosis. Findings have confirmed that endothelial cell apoptosis can regulate the imbalance of inflammation. However, compared with the above studies, the limitation of this study is that it was only verified at the cell line level, and the results were not verified in animal models. The next step will be to further verify the effectiveness of ISL using in vivo experiments.

As shown in Figure 4, the protective effect of ISL on phlebitis induced by AM and angiogenesis was verified through lumen formation experiment, PCR, and WB from the formed vessels and the expression of VEGFA. The vasculogenesis experiment reflects the early process of capillaries and is the most complete indicator of endothelial cell function in vitro. Abnormal vasculogenesis is the basis of various pathological diseases.^22^ In this study, we found that vasculogenesis in the AM group was reduced. This may be related to the damage of endothelial cells caused by AM, which causes the endothelial cells damaged by AM to not generate blood vessels normally, thus inducing venous inflammation. It is consistent with the finding that the destruction of the endothelial barrier and the reduction of angiogenesis can cause sepsis and acute respiratory distress syndrome.^23^ At the same time, studies have shown that ISL promotes vasculogenesis in HUVEC.^24^ In our study, when AM was pretreated with ISL, the number of vasculogenesis increased significantly. VEGFA is an angiogenic factor, which can increase vascular permeability and maintain vascular stability. RIPK3 regulates the expression of growth factor receptors in HUVEC to support vasculogenesis.^25^ This study found that ISL could regulate the expression of VEGFA factor to support lumen formation.

As shown in Figure 5, when AM acted on HUVEC, phosphorylated p‐NF‐κBp65 was increased. This suggests that the drug may stimulate the blood vessel wall more, damage the HUVEC, increase the permeability of blood vessels, thus leading to the upregulation of the expression of inflammatory factors.^26^ AM could activate the NF‐κB pathway in human umbilical vein endothelial cells. ISL could downregulate p‐NF‐κBp65 expression. This suggests that the protective mechanism of ISL on phlebitis induced by AM may be partly through the NF‐κB signal pathway. NF‐κB is one of the most important nuclear transcription factors in mammalian cells. The NF‐κB signal pathway is a classical anti‐inflammatory pathway. Induced by many factors, the NF‐κB dimer (p50, p65) undergoes nuclear translocation, thus causing the expression of target genes. The results of this study are consistent with that of quercetin,^27^ which by downregulation of the NF‐κB signal pathway inhibits TNF‐α incuced inflammatory response.^28^ The study of the signal pathway in this study is unitary, and the expression of NF‐κBp65, p‐NF‐κBp65 cytokine expression should be studied further. Additionally, improvements in methods are needed, such as using the WB experiment to further verify the expression of inflammatory factors and adhesion factors.

A limitation of this study is that it starts from in vitro research. We only discussed findings from the cell line and these were not further verified using animal experiments. It is suggested that the next step of the experimental study will be to consider the combination of ISL and nano materials to prepare hydrocolloids, and further explore the protective effect of ISL on phlebitis in the rabbit ear vein model. This can provide more accurate and scientific reference for clinical practice.

Previously, researchers studied chronic inflammatory diseases in clinical practice, while this study focused on acute inflammation in the nursing field. This is also the innovation of this study. This shows that ISL has significance in the prevention and treatment of clinical phlebitis and can be used as a potential therapeutic drug to prevent phlebitis. At the same time, this study can enrich nurses’ understanding of the prevention and treatment mechanism of phlebitis.

## AUTHOR CONTRIBUTIONS

**Jin‐Li Guo**: Data curation; Formal analysis; Funding acquisition; Validation; Writing—original draft; Writing—review & editing. **Xiang Han**: Data curation; Formal analysis; Writing—original draft; Writing—review & editing. **Xian‐Yan Yan**: Data curation; Formal analysis; Software; Writing—review & editing. **Juan‐Juan Wang**: Conceptualization; Data curation; Supervision; Writing—original draft; Writing—review & editing. **Ya‐Qiong Chang**: Data curation; Formal analysis; Investigation; Methodology; Project administration; Writing—review & editing. **Bei‐Lei Zhang**: Formal analysis; Project administration; Resources; Validation; Visualization; Writing—review & editing. **Xiu‐Juan Guo**: Data curation; Formal analysis; Supervision; Validation; Writing—review & editing.

## COMPETING INTERESTS

The authors declare that they have no competing interests.

## ETHICS STATEMENT

An ethics statement was not required for this study type, no human or animal subjects or materials were used.

## Data Availability

All data generated or analysed during this study are included in this article. Further enquiries can be directed to the corresponding author.
